# Efficient Medical Image Segmentation in Multisensor Imaging: A Survey in the Era of Mamba and Foundation Models

**DOI:** 10.3390/s26082558

**Published:** 2026-04-21

**Authors:** Xiu Shu, Youqiang Xiong, Zhangli Ma, Xinming Zhang, Di Yuan

**Affiliations:** 1 School of Computer Science and Cyber Engineering, Guangzhou University, Guangzhou 510006, China; shuxiu@gzhu.edu.cn; 2 Guangzhou Institute of Technology, Xidian University, Guangzhou 510555, China; 24181214212@stu.xidian.edu.cn (Y.X.); 25183224670@stu.xidian.edu.cn (Z.M.); 3 School of Science, Harbin Institute of Technology, Shenzhen 518055, China; xinmingxueshu@hit.edu.cn

**Keywords:** multisensor medical imaging, multimodal image segmentation, efficient medical image segmentation, State Space Models (Mamba), foundation models (SAM), lightweight architectures, data-efficient learning

## Abstract

Deep learning has revolutionized medical image segmentation; however, the clinical deployment of state-of-the-art models is severely impeded by their quadratic computational complexity and substantial resource demands, particularly in multisensor and multimodal imaging scenarios. In response, the field is undergoing a paradigm shift towards efficiency, characterized by the rise of linear-complexity architectures and the optimization of foundation models. This paper presents a comprehensive survey of efficient medical image segmentation methodologies, systematically reviewing the evolution from heavy, accuracy-driven models to lightweight, deployment-ready paradigms. In particular, we highlight the growing importance of efficient segmentation in multisensor medical imaging, where heterogeneous data sources such as CT, MRI, ultrasound, and infrared imaging introduce additional challenges in scalability and computational cost. We propose a novel taxonomy that categorizes these advancements into four distinct streams: (1) Mamba and State Space Models, which leverage selective scanning mechanisms to achieve global receptive fields with linear complexity; (2) Efficient Adaptation of Foundation Models, focusing on parameter-efficient fine-tuning and knowledge distillation to tailor the Segment Anything Model (SAM) for medical domains; (3) Advanced Lightweight Architectures, covering the resurgence of large-kernel CNNs and the emergence of Kolmogorov–Arnold Networks (KANs); and (4) Data-Efficient Strategies, including semi-supervised and federated learning to address annotation scarcity. Furthermore, we conduct a rigorous comparative analysis of representative algorithms on mainstream benchmarks, providing a granular evaluation of the trade-offs between segmentation accuracy and computational overhead. The survey also discusses key challenges in multisensor and multimodal settings, including modality heterogeneity, data fusion complexity, and resource constraints. Finally, we identify critical challenges and outline future research directions, serving as a roadmap for the development of next-generation efficient and scalable medical image analysis systems.

## 1. Introduction

**Figure 1:**
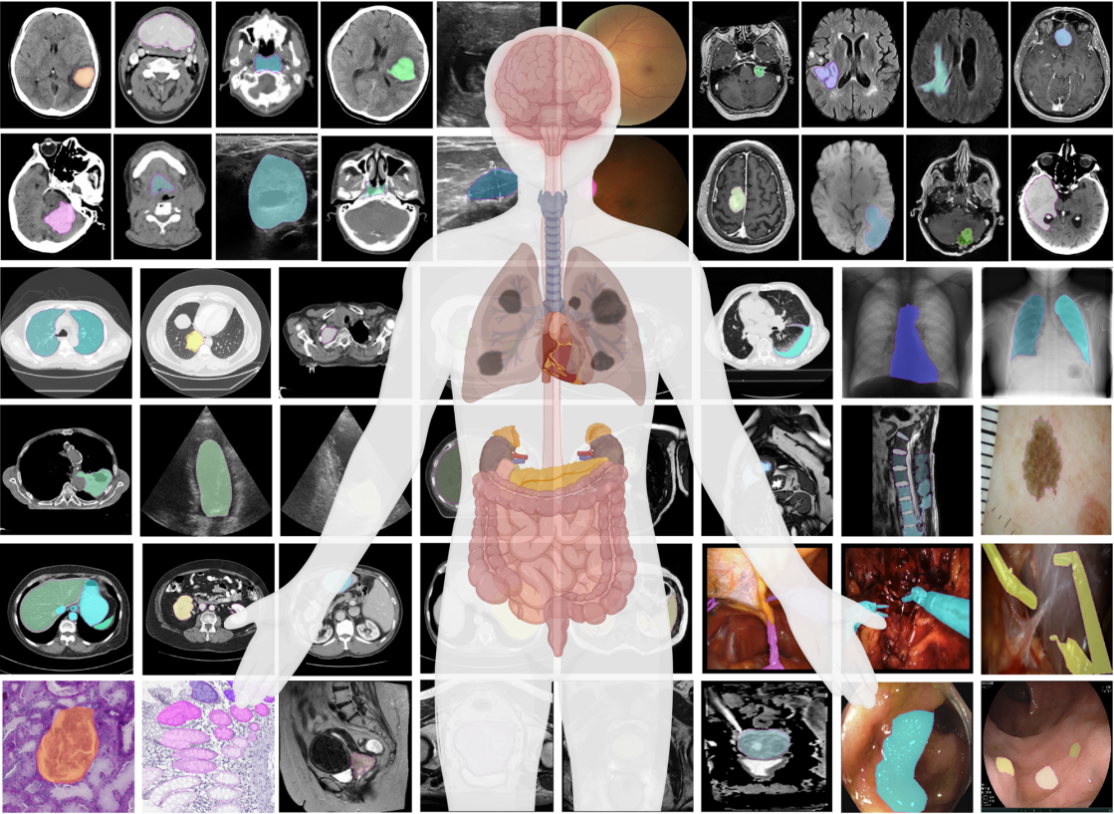
Overview of medical image segmentation tasks across diverse imaging modalities. The figure demonstrates the capability of modern foundation models (e.g., MedSAM [9]) to handle a variety of anatomical structures and pathological conditions in CT, MRI, and other modalities. Reproduced from [9] under the Creative Commons Attribution (CC BY 4.0) license.

Medical image segmentation, defined as the pixel-level identification of anatomical structures or pathological lesions from raw imaging data, constitutes a pivotal component of modern precision medicine [1,2,3]. It serves as a prerequisite for numerous downstream clinical workflows, including computer-aided diagnosis (CAD) [4,5], quantitative volumetric analysis, image-guided surgery, and radiotherapy treatment planning [6,7,8]. Driven by advancements in acquisition technologies, medical imaging data across diverse modalities, ranging from Computed Tomography (CT) and Magnetic Resonance Imaging (MRI) to microscopy, endoscopy, and infrared imaging, is being generated at an unprecedented scale, forming typical multisensor and multimodal imaging scenarios. As illustrated in Figure 1, these modalities encompass a wide spectrum of anatomical targets and pathological conditions [9]. The heterogeneous nature of multisensor data, including differences in resolution, contrast mechanisms, and noise characteristics, further increases the complexity of data processing and analysis. Consequently, the development of automated, accurate, and robust segmentation algorithms has become an imperative research direction, particularly for handling large-scale multisensor data, alleviating the labor-intensive workload of clinicians and mitigating inter-observer variability [10,11,12].

Over the past decade, the field has undergone a paradigm shift driven by deep learning, with Convolutional Neural Networks (CNNs) emerging as the *de facto* standard. The U-Net architecture, distinguished by its symmetric encoder–decoder design and semantic-preserving skip connections, has established itself as a dominant baseline [13]. Its success catalyzed numerous architectural innovations, such as V-Net [14] for volumetric data and U-Net++ [15] for nested feature aggregation. As analyzed in [16], CNN-based methodologies excel at extracting hierarchical local features and maintaining translation invariance. However, they face inherent limitations in handling global semantic information [17,18,19]. Due to the inductive bias of the convolution operation, specifically its restricted local receptive field, CNNs struggle to model explicit long-range dependencies [20,21], impeding the effective understanding of global context in high-resolution volumetric data.

To surmount the intrinsic locality of CNNs, the computer vision community witnessed a surge in adopting Transformer-based architectures. By leveraging the self-attention mechanism, Vision Transformers (ViTs) excel at capturing long-range global interactions, establishing new state-of-the-art benchmarks [22]. However, despite these performance gains, the practical deployment of ViTs in clinical scenarios faces a severe bottleneck: *Efficiency*. The standard self-attention mechanism suffers from quadratic computational complexity ($O(N^{2})$) with respect to the input sequence length. This drawback becomes prohibitive when processing high-resolution 3D medical volumes or deploying models on resource-constrained edge devices. Furthermore, Transformers typically lack the inductive bias inherent to CNNs, necessitating massive amounts of annotated training data to converge, a requirement that fundamentally contradicts the data-scarce nature of the medical domain [23,24,25].

Consequently, a critical research imperative has emerged: *How to achieve the global modeling capability of Transformers while maintaining the linear complexity and efficiency of CNNs?* In response, the field has recently shifted towards a new paradigm centered on *Efficiency* and *Generalizability*. This shift is primarily driven by three complementary directions. First, the introduction of the *Mamba* architecture [26], based on Selective SSMs, offers a compelling solution to the efficiency–accuracy trade-off by achieving global receptive fields with linear computational complexity (O(N)). Second, the release of the *Segment Anything Model (SAM)* [27] marked the arrival of the foundation model era; recent research has pivoted towards *efficient adaptation*, utilizing knowledge distillation and lightweight adapters to transfer SAM’s capabilities to medical tasks with minimal computational cost [28]. Finally, to address the prohibitive cost of expert annotation, *Data-Efficient Strategies* such as semi-supervised and federated learning have gained prominence, enabling robust model training under limited supervision while preserving data privacy [29,30].

While several reviews have covered the eras of CNNs and early Transformers [31,32], the rapid evolution of Mamba and Foundation Models remains systematically unexplored. Existing surveys typically provide summaries of traditional deep learning methods but predate this recent efficiency revolution. Other contemporary reviews focus solely on the application of SAM without addressing the emerging State Space Models or the broader landscape of lightweight architecture design [33]. To bridge this gap, this paper presents a comprehensive and systematic survey of the latest advancements in efficient medical image segmentation. The primary contributions of this work are summarized as follows:

- **Novel Efficiency-Centric Taxonomy:** We propose the first hierarchical taxonomy for the post-Transformer era, categorizing methodologies into four streamlined tracks: State Space Models (Mamba), Efficient Foundation Model Adaptation (SAM), Advanced Lightweight Architectures (incorporating KANs), and Data-Efficient Strategies.
- **In-Depth Theoretical Analysis:** We dissect the theoretical shift from quadratic-complexity Transformers to linear-complexity Mamba for volumetric modeling. Furthermore, we critically evaluate the trade-offs between zero-shot generalization and computational overhead in adapting Foundation Models (SAM) to medical domains.
- **Future Roadmap:** We identify open challenges and forecast emerging frontiers, highlighting the potential of Generative Segmentation (Diffusion Models), the rise of Kolmogorov–Arnold Networks (KANs) as successors to MLPs, and pathways for clinical deployment on resource-constrained devices.

## 2. Related Work

Before the recent paradigm shift towards State Space Models and Foundation Models, medical image segmentation was defined by the evolution of Convolutional Neural Networks (CNNs) and the subsequent adoption of Vision Transformers (ViTs). This section reviews these two dominant paradigms, identifying their structural characteristics and inherent efficiency bottlenecks. Furthermore, we analyze existing literature reviews to pinpoint the research gaps that necessitate this comprehensive survey on efficient architectures.

### 2.1. Evolution of U-Net and CNN Variants

Since the advent of deep learning, U-Net [10] has established a dominant position in medical image analysis. Its symmetric encoder–decoder architecture became the standard for preserving spatial details, seamlessly extending to volumetric data through models like V-Net [14] and 3D U-Net [34]. Concurrently, general computer vision advancements, such as DeepLabV3+ [35], influenced medical designs like MPUNet [36] by introducing atrous spatial pyramid pooling.

Subsequent research focused on enhancing feature representation through structural innovations. U-Net++ [15] introduced nested dense skip pathways to bridge the semantic gap between the encoder and decoder, while ResUNet-a [37] and R2U++ [38] integrated residual connections and recurrent units to facilitate deeper network training. To refine focus on target regions, Attention U-Net [39] embedded attention gates, whereas U-Net v2 [40] and NU-net [41] optimized feature fusion strategies. Additionally, DNetUnet [42] combined dense blocks with semi-supervised learning to improve generalization.

Parallel to accuracy improvements, significant efforts were directed toward computational efficiency. Architectures like Half-UNet [43] and ELU-Net [44] streamlined feature extraction through channel unification and deep skip connections, respectively. DRU-Net [45] integrated ResNet and DenseNet strengths to reduce parameter counts, while other works focused on specific application-oriented efficiency [46,47,48]. To mitigate the high cost of 3D convolutions, Yu et al. [49] proposed a 2.5D strategy, and Ali et al. [50] explored hardware-specific optimizations. Despite these successes, CNNs suffer from an inherent limitation: The locality of convolution restricts the receptive field, hindering the modeling of explicit global dependencies required for complex anatomical segmentation.

### 2.2. Rise and Bottlenecks of Vision Transformers

The introduction of Vision Transformers (ViTs) addressed the locality limitations of CNNs by leveraging Self-Attention mechanisms to capture global, long-range dependencies. Hybrid architectures pioneered this integration, with TransUNet [20] embedding Transformer layers within the CNN bottleneck, a concept further advanced by TransUNet+ [51]. To achieve superior feature fusion, TransFuse [52] and CoTr [53] proposed parallel and bridging strategies, while TransBTS [54] effectively applied these principles to volumetric tumor segmentation. Furthermore, UTNet [55] and HCT-Net [56] explored efficient hybrid designs via neural architecture search.

Subsequently, pure Transformer-based U-shaped architectures emerged. Swin-Unet [22] adapted the hierarchical Swin Transformer to medical imaging, an approach extended to 3D by SwinUNETR [57]. Concurrently, frameworks such as nnFormer [58] and UNesT [59] optimized the interleaving of local and global blocks for volumetric data. Advanced hierarchical designs were introduced in HiFormer [60], H2Former [61], MT-UNet [62], and SwinPA-Net [63], while DS-TransUNet [64] improved performance via dual-scale encoding.

However, the global modeling capability of Transformers incurs significant computational cost due to the quadratic complexity ($O(N^{2})$) of self-attention. Although variants like Medical Transformer [65] and LeViT-UNet [66] proposed efficient attention mechanisms and faster encoders, the heavy computational burden remains a bottleneck. While attempts like DCSAU-Net [67], MISSFormer [68], and TSE DeepLab [69] sought to balance performance and cost, the “accuracy–efficiency trade-off” remains prohibitive for high-resolution 3D data. This limitation has motivated the recent exploration of linear-complexity architectures, such as Mamba.

### 2.3. Relation to Prior Surveys

While foundational surveys [16,31,70,71] and Transformer-focused reviews [32,72] comprehensively cover CNN and self-attention architectures, they are largely representative of the traditional high-complexity deep learning era and largely overlook the recent paradigm shift towards efficiency. Our survey specifically fills this void by transitioning from general accuracy-driven methodologies to the emerging linear-complexity models like Mamba that challenge the quadratic complexity of Transformers. Furthermore, while existing works on Foundation Models [33,73] primarily treat SAM as a fixed benchmarking tool, this paper introduces a first-of-its-kind systematic categorization of efficient adaptation techniques, such as knowledge distillation, adapters, and prompting, alongside novel lightweight paradigms like KAN, which represents a fundamental shift in medical AI essential for resource-constrained clinical deployment.

To the best of our knowledge, there is currently no survey that holistically analyzes these emerging architectures under the unified theme of “Efficiency.” By providing a clear technical distinction from previous era-centric reviews, this article bridges this gap with a novel taxonomy of Mamba architectures, efficient foundation model adaptations, and advanced lightweight designs, offering a forward-looking critical roadmap for the post-Transformer efficiency-driven era of medical image analysis.

While foundational surveys [16,31,70,71] and Transformer-focused reviews [32,72] comprehensively cover CNN and self-attention architectures, they largely overlook the recent paradigm shift towards efficiency. Specifically, they fail to address emerging linear-complexity models like Mamba that challenge the quadratic complexity of Transformers. Furthermore, existing works on Foundation Models [33,73] primarily treat SAM as a fixed benchmarking tool. They lack a systematic categorization of efficient adaptation techniques, such as knowledge distillation, adapters, and prompting, and novel lightweight paradigms like KAN, which are essential for resource-constrained clinical deployment.

To the best of our knowledge, there is currently no survey that holistically analyzes these emerging architectures under the unified theme of “Efficiency.” This article bridges this gap by providing a systematic overview and a novel taxonomy of Mamba architectures, efficient foundation model adaptations, and advanced lightweight designs, offering a critical roadmap for the post-Transformer era of medical image analysis.

## 3. Methodologies for Efficient Medical Image Segmentation

**Figure 2:**
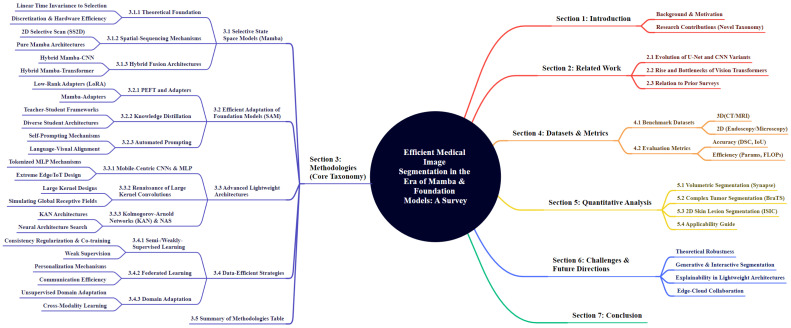
Overview of the proposed taxonomy for Efficient Medical Image Segmentation methodologies. To address the prohibitive computational costs of traditional deep learning in medical imaging, recent research has shifted towards optimizing the accuracy–efficiency trade-off.

The paradigm shift from accuracy-centric to efficiency-oriented design has catalyzed a diverse array of architectural innovations and learning strategies in medical image segmentation. Contemporary research increasingly prioritizes optimizing the trade-off between computational resource consumption (e.g., FLOPs, memory footprint) and segmentation performance. To provide a systematic analysis of this rapidly evolving landscape, we categorize existing efficient methodologies into four distinct yet interconnected streams, as illustrated in Figure 2.

First, Section 3.1 explores State Space Models (SSMs), with a focus on the Mamba architecture. These methods circumvent the quadratic complexity bottleneck of Transformers, offering a linear-complexity alternative for modeling long-range volumetric dependencies. Second, Section 3.2 investigates the Efficient Adaptation of Foundation Models, scrutinizing how large-scale models like the Segment Anything Model (SAM) are tailored for medical domains via knowledge distillation, lightweight adapters, and prompt engineering. Third, Section 3.3 reviews Advanced Lightweight Architectures, covering the resurgence of large-kernel CNNs and emerging KANs, which prioritize parameter efficiency for edge computing. Finally, Section 3.4 discusses Data-Efficient Strategies, such as semi-supervised and federated learning, which address efficiency by mitigating annotation burdens and communication overheads.

### 3.1. Selective State Space Models (Mamba): The Linear Complexity Revolution

While Vision Transformers (ViTs) have revolutionized global context modeling via Self-Attention mechanisms, their computational complexity scales quadratically with image size ($O(N^{2})$). This limitation creates severe memory bottlenecks, particularly when processing high-resolution volumetric medical data. To address this challenge, Structured State Space Models (SSMs) have been revisited as a linear-complexity alternative. Among them, the **Selective State Space Model**, popularized as *Mamba* in [26], represents a transformative breakthrough. By introducing a **Selective Scan Mechanism**, Mamba achieves global receptive fields with **linear complexity (O(N))**. This section systematically reviews the adaptation of selective SSMs for medical imaging, progressing from theoretical foundations to spatial scanning mechanisms, and finally to advanced hybrid architectures.

#### 3.1.1. Theoretical Foundation: From Linear Time Invariance to Selection

State Space Models (SSMs) conceptually map a 1-D input sequence x(t)∈R to an output y(t)∈R through a latent state $h\left(t\right)\in R^{N}$. In the context of deep learning, these models are inspired by continuous-time systems governed by the linear Ordinary Differential Equation (ODE), as formulated in foundational vision-SSM works [26,74,75]:

(1) $$h^{\prime }\left(t\right)=Ah\left(t\right)+Bx\left(t\right),\;y\left(t\right)=Ch\left(t\right)$$

where $A\in R^{N\times N}$ denotes the evolution matrix, and B,C are projection parameters. To be computationally feasible on digital hardware for tasks like medical segmentation, this continuous system is discretized using the Zero-Order Hold (ZOH) method with a timescale parameter Δ, a process detailed in U-Mamba [76]. The discretized recurrence is formulated as

(2) $$h_{t}=\bar{A}h_{t-1}+\bar{B}x_{t},\;y_{t}=Ch_{t}$$

where $\bar{A}=\exp\left(\Delta A\right)$ and $\bar{B}={\left(\Delta A\right)}^{-1}\left(\exp\left(\Delta A\right)-I\right)\cdot \Delta B$.

Traditional SSMs are **Linear Time Invariant (LTI)**, implying that the matrices A,B,C remain fixed regardless of the input. However, as highlighted in [77,78], LTI models struggle to perform content-aware compression of context, which is critical for distinguishing lesions from complex anatomical backgrounds. Mamba addresses this limitation by introducing a **Selection Mechanism**, where parameters B,C,Δ are derived directly from the input $x_{t}$ (i.e., $B(x_{t})$, $\Delta (x_{t})$).

**Figure 3:**
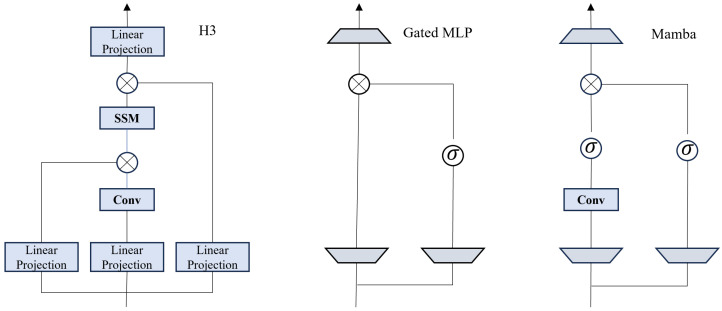
Architecture of the Selective State Space Model (Mamba) [26]. Schematically redrawn and adapted from [26]. Unlike traditional H3 or MLP blocks, the Mamba block (Right) introduces a **Selection Mechanism** where the parameters B, C, and Δ are computed directly from the input *x* via linear projections. This input-dependent dynamic allows the model to selectively compress context with linear complexity O(N).

As explicitly illustrated in Figure 3, the Mamba architecture diverges from traditional designs. Unlike standard H3 or MLP blocks which use static parameters, the Mamba block (shown on the Right of the figure) derives the parameters B, C, and Δ directly from the input *x* via linear projections (denoted as $s_{B},s_{C}$ in the diagram). This makes the system dynamic and content-aware. This mechanism allows the model to selectively propagate relevant medical features while suppressing irrelevant noise, achieving **linear computational complexity (O(N))**. This efficiency advantage over the quadratic complexity of Transformers ($O(N^{2})$) has been empirically validated in high-resolution segmentation tasks [79,80]. Furthermore, similar linear-complexity characteristics are explored in Recurrent Neural Network (RNN) variants like RWKV [81,82], reinforcing the trend towards efficient sequence modeling in medical imaging.

#### 3.1.2. Spatial-Sequencing: Scanning Mechanisms and Pure Architectures

Since Mamba is inherently designed for 1-D causal sequences, a critical challenge in medical imaging is adapting it to non-causal 2D/3D spatial data without compromising local continuity.

**Figure 4:**
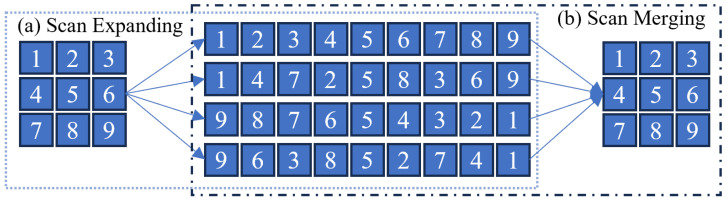
The 2D Selective Scan (SS2D) Mechanism used in Pure Mamba Architectures [83]. Schematically redrawn and adapted from [83]. (**a**) The input feature map is flattened into sequences along four distinct directions to traverse the spatial domain. (**b**) Each sequence is processed independently by SSMs and then merged to restore the 2D spatial structure, ensuring global context modeling without breaking spatial logic.

**The SS2D Mechanism:** To bridge this gap, pioneer works such as VM-UNet [83] and U-Mamba [76] introduced the **Visual State Space (VSS) Block**, equipped with a **2D Selective Scan (SS2D)** mechanism (also referred to as the Cross-Scan Module). As shown in Figure 4, this mechanism flattens the image features Z along four distinct directions (top-left to bottom-right, bottom-right to top-left, etc.) to model global dependencies. The aggregated output feature $Z_{out}$ is computed by summing the processed sequences from all scanning directions:

(3) $$Z_{out}=\sum_{i=1}^{4}\mathrm{norm}\left(\mathrm{SSM}\left({\mathrm{scan}}_{i}\left(Z_{in}\right)\right)\right)$$

This multi-directional scanning strategy ensures that each pixel integrates information from all neighbors linearly, effectively simulating the global receptive field of Vision Transformers.

**Pure Mamba Architectures:** Leveraging this mechanism, several studies have proposed U-shaped networks constructed entirely from Mamba blocks to replace CNNs or ViTs. VM-UNet++ [84] and Mamba-UNet [80] utilize VSS blocks for both encoding and decoding stages, establishing a baseline that significantly reduces FLOPs while maintaining segmentation accuracy. For volumetric data, SegMamba-V2 [85] extends the scanning strategy to a tri-oriented approach to capture inter-slice correlations in 3D space. To further improve scanning adaptability for organs with irregular shapes, Switch-UMamba [86] proposes a dynamic routing mechanism, while LoG-VMamba [87] and H-vmunet [88] introduce local–global and high-order interaction strategies to better preserve spatial coherency.

#### 3.1.3. Hybrid Fusion and Advanced Feature Interaction

While pure Mamba models excel in efficiency, they may lack the strong local inductive bias of CNNs or the robust channel-mixing capabilities of Transformers. Consequently, a plethora of hybrid architectures have been proposed to synergize the strengths of multiple paradigms.

**Figure 5:**
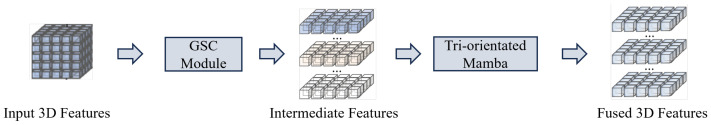
Overview of a representative Hybrid Mamba–CNN Architecture (e.g., SegMamba [89]). Schematically redrawn and adapted from [89]. The framework typically employs Convolutional layers for local feature extraction and downsampling, while integrating Mamba blocks in the bottleneck or encoder stages to capture long-range global dependencies (Equation (4)), combining the strengths of both inductive bias and global receptivity.

**Hybrid Mamba-CNN Architectures:** The majority of recent works adopt a paradigm that integrates Convolution for local feature extraction and Mamba for global modeling. A representative design is SegMamba [89], which incorporates a **Gated Spatial Convolution (GSC)** before the SSM layer to enhance local feature representation and suppress noise. The feature fusion process in such hybrid blocks can be formulated as

(4) $$Z_{GSC}={\mathrm{Conv}}_{3\times 3}\left(Z_{in}\right)⊙\sigma \left({\mathrm{Conv}}_{1\times 1}\left(Z_{in}\right)\right)+Z_{in},$$

(5) $$Y=\mathrm{SSM}\left(Z_{GSC}\right)+Z_{GSC}$$

where ⊙ denotes element-wise multiplication, and σ represents the activation function. As depicted in Figure 5, this GSC module effectively filters local features before long-range modeling. Similar hybrid strategies are adopted in MambaVesselNet++ [90] and SegUMamba [91], which embed Mamba blocks into the bottlenecks of U-Net variants.

To handle multi-scale features effectively, specialized multi-scale fusion modules have been introduced in MSCD-VM-UNet [92], MSM-UNet [93], and MSVM-UNet [94]. LKM-UNet [78] combines Mamba with large-kernel convolutions to maximize receptive fields. Dual-encoder designs are utilized in DCM-Net [95] and CMHNet [96] to process local and global information in parallel branches, while CNS-UNet [97] and GL-MambaNet [98] focus on efficient feature fusion mechanisms. Additionally, SegEO Mamba [99] specifically optimizes the decoder stage with Mamba blocks to enhance reconstruction quality.

**Hybrid Mamba-Transformer & Advanced Variants:** Some works explore the synergy between Mamba and other domains such as Attention or Frequency analysis. Swin-UMamba [100] leverages ImageNet-pretrained weights to enhance generalization, while HMT-UNet [101] and HybridMamba [102] fuse Mamba with Transformer blocks to balance complexity and accuracy. Furthermore, FMamba [103] and EM-Net [104] incorporate frequency domain information to improve boundary detection capabilities.

Notable variants focusing on lightweight deployment include LightM-UNet [105], Light-VM2D-UNet [106], LVMC-UNet [107], and MAFUNet [108]. Novel applications include UD-Mamba [109] for uncertainty estimation and Weak-Mamba-UNet [110] and DCMamba [111] for weakly-supervised and semi-supervised settings. Additionally, InceptionMamba [112] demonstrates the versatility of Mamba in microscopic segmentation tasks.

**Critical Discussion on SSMs:** While Mamba provides a breakthrough in modeling long-range dependencies with linear complexity, its transition from 1D causal sequences to 2D/3D medical images introduces a “spatial discontinuity” risk. Our analysis of the literature suggests that Mamba’s performance is highly sensitive to the scanning trajectory; unlike the inherent translation invariance of CNNs, SSMs lack a natural multi-dimensional inductive bias. Furthermore, while the linear complexity reduces the memory bottleneck for high-resolution volumes, the recurrent nature of state updates can lead to “long-term forgetting” in extremely long sequences (e.g., whole-body CT), necessitating more complex memory-management architectures.

### 3.2. Efficient Adaptation of Foundation Models: Bridging the Efficiency Gap

The advent of Foundation Models, exemplified by the Segment Anything Model (**SAM**) [27], has inaugurated a paradigm of “promptable segmentation” characterized by robust zero-shot generalization. However, the direct deployment of standard SAM in medical imaging faces two primary impediments: **Domain Shift**, stemming from the significant distributional disparity between natural training images and diverse medical modalities; and **Computational Inefficiency**, driven by the prohibitive resource demands of the heavy ViT-H backbone. To bridge this gap, contemporary research has established a systematic framework for efficient adaptation. This section taxonomizes these methodologies into three interconnected streams: Parameter-Efficient Fine-Tuning (PEFT), Knowledge Distillation, and Automated Prompting.

#### 3.2.1. Parameter-Efficient Fine-Tuning (PEFT) and Adapters

Full fine-tuning of billion-parameter models is often computationally intractable and prone to catastrophic forgetting. PEFT strategies mitigate this by freezing the pre-trained backbone weights $W_{0}$ and optimizing only a small set of additive parameters. The prevailing approach involves inserting lightweight **Adapter** modules into the Transformer blocks to inject domain-specific knowledge.

**Mechanism:** A representative approach, MA-SAM [113], mathematically formulates this adaptation process using low-rank matrices. For an input feature $x\in R^{d}$, the output y of an adapted layer is defined as

(6) $$y=W_{0}x+s\cdot B\left(\sigma \left(Ax\right)\right),$$

where $A\in R^{r\times d}$ serves as a down-projection matrix, $B\in R^{d\times r}$ as an up-projection matrix (with rank r≪d), σ denotes a non-linear activation function, and *s* is a learnable scaling factor. This bottleneck structure allows the model to learn medical-specific features, such as 3D volumetric context, with minimal parameter overhead. Further exploring this direction, De-LightSAM [114] extends the framework by decoupling modality-specific knowledge, while DAFT [115] proposes a dynamic adjustment of fine-tuning strategies based on data distribution.

**Structural Innovations:** Recent works have innovated the internal architecture of adapters beyond standard Multi-Layer Perceptrons (MLPs). The 3DSAM-adapter [116] introduces a holistic modification scheme to effectively transfer 2D priors to 3D volumetric data. Notably, drawing parallels to the efficiency of State Space Models discussed in Section 3.1, Mamba-SAM [117] and MambaSAM Framework [118] innovatively propose the Mamba-Adapter.

**Figure 6:**
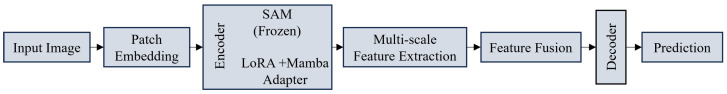
Architecture of the Mamba-SAM Adapter [117]. Schematically redrawn and adapted from [117].

As specifically illustrated in Figure 6, the Mamba-SAM architecture freezes the massive image encoder (shown in Blue) and integrates a lightweight Encoding Mamba Adapter **(EMA)** (shown in Orange). Unlike the matrix decomposition in Equation (6), these approaches leverage the linear complexity of SSMs to model long-range dependencies within the frozen ViT backbone:

(7) $$y_{mamba}=x+\lambda \cdot \mathrm{SSM}\left(\mathrm{Norm}\left(x\right)\right)$$

Compared to attention-based adapters, this design offers a superior trade-off between adaptation accuracy and computational cost by avoiding quadratic complexity in the adapter branch.

#### 3.2.2. Knowledge Distillation and Model Compression

**Figure 7:**
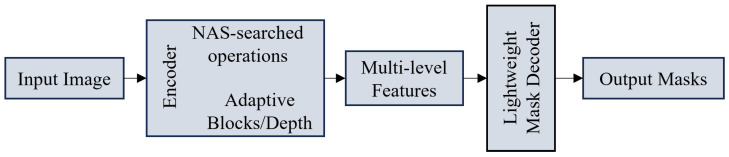
The Knowledge Distillation Pipeline for Efficient SAM [119]. Schematically redrawn and adapted from [119]. A frozen Heavy Teacher (SAM-ViT-H) guides a Lightweight Student (e.g., CNN or ViT-Tiny). The process involves minimizing feature discrepancy at multiple stages (Equation (8)), enabling the student to inherit the teacher’s semantic representations while maintaining high inference speed on edge devices.

While adapters significantly reduce training costs, the inference latency imposed by the heavy backbone remains a challenge for real-time applications. **Knowledge Distillation (KD)** addresses this by transferring the capabilities of a heavy “Teacher” (e.g., SAM-ViT-H) to a lightweight “Student” network. As shown in Figure 7, the student network is trained to mimic the teacher’s hierarchical features and segmentation masks.

**Distillation Frameworks:** As proposed in EfficientMedSAM [119], the training objective typically combines segmentation loss with hierarchical feature alignment:

(8) $$L_{total}=L_{seg}\left(P_{S},Y_{GT}\right)+\alpha \sum_{l}{∥F_{T}^{l}-\phi \left(F_{S}^{l}\right)∥}^{2},$$

where $F_{T}^{l}$ and $F_{S}^{l}$ denote feature maps from the *l*-th layer of the Teacher and Student, respectively, and ϕ represents a projection layer aligning the student’s channel dimensions to the teacher’s. This formulation ensures that the student inherits the rich semantic representation of the foundation model. Works such as KD-MedSAM [120] and Distilled-SAM [121] specifically focus on distilling the image encoder, which constitutes the primary computational bottleneck.

**Diverse Student Architectures:** To maximize deployment efficiency, various student backbones have been explored. LWUSAM [122] and ERViTT [123] employ tiny ViTs combined with quantization techniques to achieve real-time speeds on CPU hardware. To leverage the inductive bias of convolutions, RepMedSAM [124] and Rep-MedSAM [125] utilize structural re-parameterization CNNs (e.g., RepVGG) as student backbones. Similarly, Med-FastSAM [126] adopts a CNN-student strategy to ensure robust domain generalization. This paradigm is further validated in KDUNet [127] and EKDSAM [128], demonstrating that lightweight students can approximate the teacher’s zero-shot performance with significantly reduced computational overhead.

#### 3.2.3. Automated Prompting and Zero-Shot Efficiency

The original SAM relies on manual prompts (e.g., clicks or bounding boxes), which is inefficient for high-throughput clinical workflows. Research in this stream aims to automate the “Prompt Engineering” phase, focusing on two primary automation pathways: **Self-Prompting** and **Language-Visual Alignment**. These strategies are discussed in detail below.

**Self-Prompting Mechanisms:** AutoSAM [129] introduces an auxiliary **Prompt Generator Network** ($E_{prompt}$). Instead of human input, this network infers sparse or dense prompts directly from image features:

(9) $$P_{auto}=E_{prompt}\left(I\right),\;M={\mathrm{SAM}}_{decoder}\left(Z_{img},P_{auto}\right)$$

This mechanism effectively converts the interactive model into a fully automatic one. SPLV [130] utilizes pixel-wise classifiers to generate initial seeds, while IASAM [131] employs SAM’s coarse predictions as iterative prompts for refinement. In semi-supervised settings, ESAMP [132] optimizes prompting policies using preference data to improve label quality.

**Language-Vision Alignment:** Integrating textual guidance offers another efficient pathway. MedCLIP-SAM [133] and MedCLIP-SAMv2 [134] leverage CLIP to align medical text descriptions with visual features, allowing users to perform segmentation via semantic queries (e.g., “segment the left ventricle”) rather than manual localization. SAMU [135] and UniverSeg [136] further explore the universality of such multi-modal prompting. Extensive benchmarks in literatures [137,138] validate that these zero-shot strategies can achieve competitive performance in data-scarce scenarios without the need for extensive retraining.

**Analytical Insights on SAM Adaptation:** The primary limitation of adapting SAM to medical tasks is the persistent “Domain Shift” and the heavy inference latency of its ViT backbone. Although PEFT strategies like LoRA and adapters significantly reduce the number of trainable parameters, they do not alleviate the computational burden of the frozen high-resolution image encoder during inference. Moreover, the segmentation quality remains heavily dependent on prompt precision (clicks or boxes), which may hinder its application in fully automated, high-throughput clinical pipelines where manual intervention is not feasible.

### 3.3. Advanced Lightweight Architectures: Beyond Standard Convolutions

While Foundation Models and Mamba have introduced novel paradigms for global context modeling, the optimization of traditional backbones remains a vibrant and essential research area, particularly for deployment in resource-constrained environments. This section reviews the evolution of lightweight architectures, transitioning from MLP-based designs and the renaissance of Large Kernel Convolutions to the emerging KAN, alongside automated design via Neural Architecture Search (NAS).

#### 3.3.1. Mobile-Centric CNNs and MLP-Based Architectures

To enable deployment on point-of-care devices (e.g., mobile ultrasound probes), researchers have sought to eliminate the quadratic complexity of Transformers while retaining their global receptive fields. A pivotal direction in this domain is the utilization of **Multi-Layer Perceptrons (MLPs)** as efficient token mixers.

**Figure 8:**
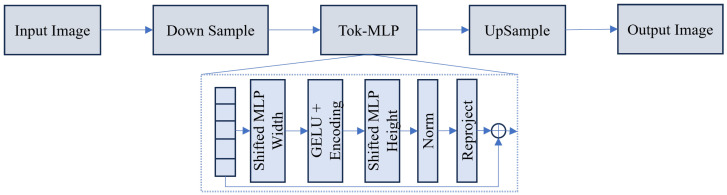
The Tokenized MLP Block in UNeXt [139]. Schematically redrawn and adapted from [139]. As shown in the diagram, the input features are shifted along channels before being processed by MLPs. This mechanism allows the network to model token interactions without the heavy matrix multiplication of Self-Attention, enabling high-speed inference on mobile devices.

**Tokenized MLP Mechanisms:** As illustrated in Figure 8, UNeXt [139] introduces a tokenized MLP block. By shifting the channels of convolutional features to mix information across tokens, it simulates the global interaction of attention with significantly fewer parameters. Mathematically, the shifted MLP operation can be expressed as

(10) $$Y=F_{\mathrm{MLP}}\left(\mathrm{Shift}\left(X\right)\right)+X,$$

where the Shift operation displaces channels along spatial dimensions to aggregate local context. Building on this hybrid philosophy, Mobile U-ViT [140] integrates mobile-friendly inverted bottleneck blocks with lightweight Vision Transformers. Furthermore, LcmUNet [141] and CNL-UNet [142] optimize the interaction between CNNs and MLPs to strike a balance between local texture extraction and global context modeling for real-time inference.

**Extreme Lightweight Design for Edge/IoT:** Targeting extreme resource constraints, several works push the limits of parameter reduction. U-Lite [143] and Mini-Net [144] demonstrate that sub-million parameter models can achieve competitive performance through the strategic use of efficient depth-wise separable convolutions. For IoT-specific deployment, EdgeMedNet [145] and LDMRes-Net [146] propose specialized residual blocks designed to maximize inference speed on edge hardware. The practical feasibility of these architectures is critically evaluated in literature [147].

**Feature Enhancement Strategies:** To mitigate the potential capacity reduction inherent in lightweight models, advanced attention and fusion mechanisms have been widely adopted. EGE-UNet [148] and MALUNet [149] utilize group-wise attention to enhance feature discrimination with minimal computational cost. Incorporating frequency domain analysis, GFUNet [150] and PMFSNet [151] efficiently capture global dependencies. Furthermore, specific optimizations for bottleneck and skip connections are explored in MixUNet [152], MAUNext [153], ESDMR-Net [154], and LeaNet [155], which refine feature aggregation. Tailored solutions for specific modalities include LAEDNet [156] and CPCANet [157], which address the low-contrast nature of ultrasound images, while PIS-Net [158] handles image distortions in portable devices. Other variants, such as IRUNet [159] and DFBU-Net [160], further demonstrate the versatility of lightweight designs across diverse clinical tasks.

#### 3.3.2. The Renaissance of Large Kernel Convolutions

A parallel stream of research posits that the success of Transformers stems primarily from their large receptive fields rather than the attention mechanism itself. Consequently, **Large Kernel (LK)** convolutions have been revisited to modernize CNN architectures.

**Large Kernel Designs:** 3D UX-Net [161] adapts the ConvNeXt design paradigm to 3D medical data, and it employs large depth-wise convolutions (e.g., kernel sizes ranging from 7×7×7 to 31×31×31) to mimic the global scope of Swin Transformer blocks. The operation is formulated as

(11) $$Y={\mathrm{PW}}_{2}\left(\mathrm{GELU}\left({\mathrm{PW}}_{1}\left({\mathrm{DWConv}}_{K\times K\times K}\left(X\right)\right)\right)\right),$$

where DWConv denotes depth-wise convolution with kernel size *K*, and PW denotes point-wise convolution. This approach is further optimized in ConvUNeXt [162] and CMUNeXt [163], which integrate large kernels into U-shaped architectures to maximize performance.

**Deformable and Efficient Transformers:** Addressing the fixed geometric structure of standard convolutions, DLKA-Net [164] introduces deformable large kernel attention, allowing the convolutional grid to adaptively deform to irregular organ shapes. In parallel, efficient Transformer variants continue to evolve by reducing redundancy. SegFormer3D [165] and Slim UNETR [166] focus on pruning redundant attention heads in volumetric processing. Hybrid approaches like SegStitch [167] combine large kernels with lightweight ViTs. Hierarchical hybrid designs in DSTUNET [168] and EMCAD [169] to improve multi-scale feature aggregation.

#### 3.3.3. Kolmogorov–Arnold Networks (KAN) and Neural Architecture Search

A paradigm-shifting development involves KANs and the automation of design via Neural Architecture Search (NAS).

**Figure 9:**
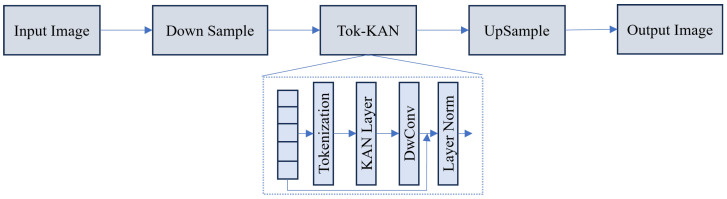
The U-KAN Architecture [170]. Schematically redrawn and adapted from [170]. Unlike traditional MLPs that use fixed activation functions on nodes, KAN layers (Equation (12)) employ learnable B-spline functions on edges. This design allows for higher accuracy with fewer parameters, making it an ideal building block for efficient medical segmentation.

**KAN Architectures:** Unlike Multi-Layer Perceptrons (MLPs) that use fixed activation functions on nodes, KANs place learnable activation functions on edges (splines). As introduced in U-KAN [170] and shown in Figure 9, a KAN layer is defined as a collection of 1-D functions $\phi_{q,p}$ parameterized as B-splines:

(12) $$\mathrm{KAN}{\left(x\right)}_{q}=\sum_{p=1}^{n_{in}}\phi_{q,p}\left(x_{p}\right)$$

This structure offers superior interpretability and parameter efficiency compared to MLPs. Recent works have rapidly integrated KANs with other efficient paradigms. KM-UNet [171] and KMUNet [172] pioneer the fusion of KANs with Mamba, leveraging KAN’s nonlinear fitting capability alongside Mamba’s sequence modeling. KANSeg [173] further validates this paradigm on multi-organ segmentation tasks.

**Neural Architecture Search (NAS) and Structural Optimization:** To automate the design of efficient models, NAS is employed to discover optimal connectivity patterns. HCT-Net [56] and BiX-NAS [174] automatically identify optimal hybrid architectures combining CNN and Transformer layers. Beyond NAS, researchers are exploring novel structural regularizations. NexToU [175] and G-CASCADE [176] incorporate topological constraints and graph convolutions to improve geometric accuracy without heavy computation. Focusing on boundary refinement and feature consistency, EANet [177], BATFormer [178], and EPT-Net [179] integrate specialized edge-perception modules. To improve training convergence, SelfReg-UNet [180], MSKD [181], and Mu-Net [182] leverage self-regularization and deep supervision strategies. Additionally, WaveFormer [183] and LMIS [184] explore wavelet-driven and multi-scale fusion techniques. Other effective variants, such as MK-UNet [185], E2ENet [186], ES-UNet [187], and DAE-Former [188], further contribute to the diversity of efficient architectures.

**Limitations of KANs:** Despite the superior parameter efficiency and interpretability of KANs, their current scalability for 3D medical imaging remains a concern. Analytically, spline-based activation functions on edges are computationally more expensive to optimize than standard MLPs during the training phase. The literature indicates potential convergence stability issues when scaling KANs to large-scale datasets, suggesting that they are currently more suitable as lightweight task-specific heads or in hybrid designs rather than as a complete backbone replacement.

### 3.4. Data-Efficient Strategies: Learning with Limited Supervision

While architectural innovations such as Mamba and KAN significantly improve computational efficiency, the prohibitive cost and expertise required for acquiring pixel-level medical annotations remain a critical bottleneck. To mitigate this reliance on large-scale labeled datasets, researchers have developed data-efficient strategies that maximize performance under label scarcity. This section reviews three pivotal paradigms: Semi-Supervised/Weakly-Supervised Learning, Federated Learning, and Domain Adaptation.

#### 3.4.1. Semi-Supervised and Weakly-Supervised Learning

Semi-Supervised Learning (SSL) aims to leverage a small set of labeled data alongside a vast volume of unlabeled data. A dominant paradigm in this domain is **Consistency Regularization**, often implemented via Teacher–Student frameworks.

**Figure 10:**
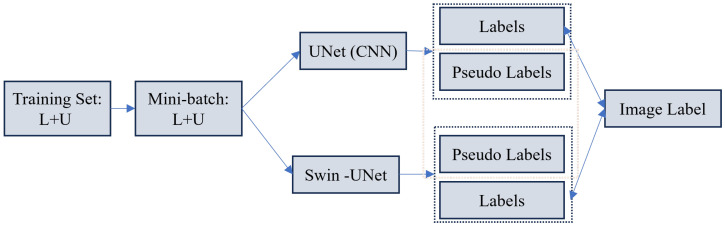
A representative semi-supervised co-training framework SSL4MIS [190]. Schematically redrawn and adapted from [190]. Two networks (e.g., CNN and Transformer/Mamba) are trained simultaneously. As shown in the diagram, they generate pseudo-labels for each other on unlabeled data (refer to Equation (13)), enforcing consistency to learn robust representations from limited annotations.

**Consistency and Co-training:** As illustrated in Figure 10, recent methodologies have evolved from simple consistency checks to dual-view co-training strategies. Furthermore, [189] proposes embedding analysis tasks into the latent space to enforce cross-space consistency, which has demonstrated superior performance in the multi-dimensional analysis of echocardiography. The SSL4MIS [190] proposes a cross-teaching mechanism where a CNN and a Transformer mutually learn from each other’s pseudo-labels. This approach effectively mitigates the confirmation bias inherent in single-model architectures. To further enhance robustness, ACT-NET [191] introduces an asymmetric co-teacher framework to balance model complexity and accuracy. Mathematically, for an unlabeled input $x_{u}$, the consistency loss $L_{cons}$ enforces the predictions from two different networks (or perturbations), $f_{1}$ and $f_{2}$, to remain invariant:

(13) $$L_{cons}=E_{x_{u}}\left[∥f_{1}\left(x_{u};\theta_{1}\right)-f_{2}\left(x_{u};\theta_{2}\right){∥}^{2}\right]$$

Advanced variations include MixSegNet [192], which fuses multiple mixed-supervisory signals, and SemiSUn [193], which addresses unpaired multi-modal learning. Notably, Semi-Mamba-UNet [29] integrates the Mamba architecture into SSL, utilizing pixel-level contrastive learning to capture long-range dependencies within unlabeled data.

**Contrastive and Uncertainty Learning:** To improve feature discrimination in low-data regimes, MCSC [194] and SimCVD [195] incorporate contrastive losses to align representations of semantically similar anatomical structures. Addressing the reliability of pseudo-labels, RSemiS [196] and USemiS [197] introduce variance reduction techniques to filter out uncertain predictions.

**Weak Supervision:** Moving beyond pixel-level dense labels, ScrS [198] and AEScr [199] demonstrate that sparse scribbles can achieve competitive performance. PA-Seg [200] pushes this limit further by utilizing extreme point annotations. To handle the label noise inherent in weak supervision, AINoL [201] and AENoL [202] propose robust learning frameworks. Additionally, few-shot regimes are explored in RMRef [203] and ADFew [204] to enable rapid adaptation to new classes.

#### 3.4.2. Federated Learning and Privacy Preservation

Federated Learning (FL) addresses the “data silo” problem by training algorithms across decentralized institutions without exchanging raw patient data. The core challenge lies in the **Non-IID (Independent and Identically Distributed)** nature of medical data across different centers.

**Figure 11:**
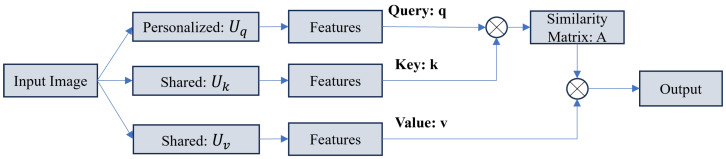
Architecture of Personalized Federated Learning [30]. Schematically redrawn and adapted from [30]. As depicted, a central server aggregates global knowledge, while local clients perform personalized updates (Equation (14)) to adapt to site-specific data distributions. This dual mechanism ensures that the model performs well on local heterogeneous data while benefiting from global knowledge.

**Personalization Mechanisms:** To handle statistical heterogeneity, FedDP [30] proposes a dual personalization mechanism. As illustrated in Figure 11, instead of maintaining a single global model, personalized local models are adapted to site-specific distributions (e.g., specific MRI scanner characteristics). The local optimization objective typically includes a proximal term to prevent the local model $\theta_{k}$ from diverging excessively from the global model $\theta_{global}$:

(14) $$\min_{\theta_{k}}L_{task}\left(D_{k};\theta_{k}\right)+\frac{\mu }{2}{∥\theta_{k}-\theta_{global}∥}^{2}$$

where $D_{k}$ denotes the local dataset of client *k*. This approach is further refined in IOP-FL [205], which distinguishes between “inside” (participating clients) and “outside” (unseen domains) personalization. FedMix [206] addresses the scenario where clients possess varying levels of annotation quality.

**Communication Efficiency:** High communication overhead prevents the deployment of large models in FL. FKD-Med [207] introduces a Knowledge Distillation (KD) framework within FL. Instead of transmitting full model parameters, clients exchange lightweight student models or logits, significantly reducing bandwidth usage while preserving privacy. Furthermore, Wu et al. [208] introduces contrastive learning into FL to align feature spaces across clients without sharing raw data. Xu et al. [209] specifically targets bridging the generalization gap between federated and centralized training.

#### 3.4.3. Domain Adaptation and Cross-Modality Learning

Clinical models often suffer from performance degradation when applied to unseen domains (e.g., different scanner vendors) or when specific imaging modalities are missing during inference.

**Figure 12:**
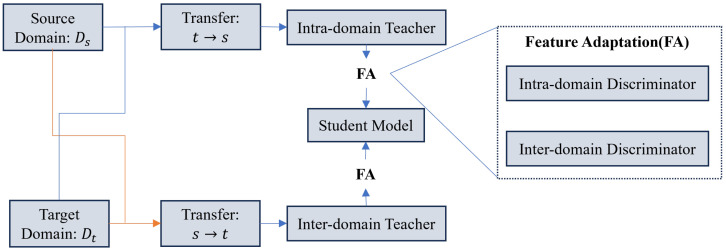
Cross-Modality Domain Adaptation Framework [212]. Schematically redrawn and adapted from [212]. The model utilizes adversarial learning (Equation (15)) or prototype alignment (Equation (16)) to transfer knowledge from a labeled Source Domain (e.g., CT) to an unlabeled Target Domain (e.g., MRI). This structure allows the network to generalize to new imaging modalities or handle missing data sequences without requiring new annotations.

**Unsupervised Domain Adaptation (UDA):** UDA aims to transfer knowledge from a labeled Source Domain (*S*) to an unlabeled Target Domain (*T*). MT-UDA [210] extends this to scenarios with limited source labels. A recent Mamba-based extension, Mamba-UDA [211], demonstrates the superior efficacy of SSMs in capturing domain-invariant global features compared to CNNs. LE-UDA [212] proposes a label-efficient framework to align feature distributions. Furthermore, recent advancements in cross-domain stylization and dual adversarial feature learning [213] highlight the importance of pixel-level distribution alignment alongside high-level semantic consistency. By integrating style transfer with edge-consistency refinement, this approach effectively preserves structural integrity and minimizes disparities in thin, texture-sensitive structures. While originally optimized for crack segmentation, these strategies of aligning low-level appearance and semantics provide valuable insights for robustly capturing domain-invariant features across disparate medical imaging sensors. As shown in Figure 12, this is often achieved via adversarial learning, where a discriminator *D* attempts to distinguish between source and target features f(x), while the segmentor aims to deceive it:

(15) $$L_{adv}=E_{x_{s}∼S}\left[\log D\left(f\left(x_{s}\right)\right)\right]+E_{x_{t}∼T}\left[\log\left(1-D\left(f\left(x_{t}\right)\right)\right)\right]$$

**Missing Modality and Multi-Domain Learning:** In multi-modal tasks (e.g., MRI with T1, T2, FLAIR), specific modalities may be unavailable. Ref. [214] utilizes knowledge distillation, training a “Teacher” on full modalities and distilling its knowledge to a “Student” with missing inputs via prototype matching:

(16) $$L_{proto}={∥P_{Teacher}-P_{Student}∥}^{2},$$

where P represents class-specific prototypes. Similarly, Rahimpour et al. [215] employs cross-modal distillation to enhance the robustness of single-sequence inference. MDViT [216] introduces domain adapters to mitigate negative transfer when handling multiple small datasets. Finally, MI-Seg [217] provides a unified framework for processing diverse modalities, ensuring consistent performance across different imaging protocols.

### 3.5. Efficient Architectures for Multisensor Data Fusion

To align with the multisensor focus highlighted in our title, we provide a dedicated analysis of architectures designed for heterogeneous data fusion (e.g., PET/CT or multi-sequence MRI). Given the significant modality gap and computational cost of multisensor imaging, recent efficiency-driven models prioritize several strategies: (1) **Lightweight Gated Fusion:** Instead of exhaustive cross-attention, models such as HMT-UNet [101] use channel-wise gating to adaptively weight complementary information from different sensors. (2) **Cross-modal SSMs:** Recent Mamba-based variants utilize the linear-complexity selective scan mechanism to interleave sequences from different modalities, enabling global feature interaction without the quadratic memory growth typical of Transformer-based fusion. (3) **Modality-Bridge Adapters:** In foundation model adaptation, lightweight adapters are inserted to bridge the domain shift between disparate sensors while keeping the primary backbone frozen. These methodologies ensure that the enhanced diagnostic power of multisensor imaging remains computationally feasible for real-time clinical workflows.

### 3.6. Summary of Methodologies

In this section, we have systematically reviewed the recent advancements in efficient medical image segmentation, categorizing them into four distinct paradigms: State Space Models (Mamba), Efficient Foundation Models (SAM), Advanced Lightweight Architectures (CNN/KAN), and Data-Efficient Strategies. The evolution of these methods demonstrates a clear trend: moving away from purely accuracy-driven, computationally heavy models (like standard ViTs) towards architectures that strike an optimal balance between performance, inference speed, and annotation efficiency.

To provide a clear overview of this rapidly evolving landscape, we summarize representative methods from each category in Table 1. This structured taxonomy highlights their core mechanisms, architectural characteristics, and publication years, serving as a guide for selecting appropriate methodologies based on specific clinical constraints such as hardware limitations or data scarcity.

Having established the methodological foundations and categorized the state-of-the-art approaches, the following sections will detail the datasets used for benchmarking and provide a comparative analysis of experimental results to quantify the efficiency-accuracy trade-offs of these architectures.

## 4. Datasets and Evaluation Metrics

The rigorous evaluation of efficient medical image segmentation methodologies relies heavily on standardized benchmarks and consistent metrics. Based on the literature reviewed in Section 3, we categorize the mainstream datasets into three primary domains: Abdominal and Cardiac Segmentation (Volumetric CT/MRI), Brain Tumor Segmentation (Volumetric MRI), and Endoscopy/Skin/Retinal Segmentation (2D RGB/Microscopy). Table 2 provides a comprehensive summary of these datasets, detailing their modality, dimensions, and target structures. Furthermore, we define the quantitative metrics used to assess both segmentation accuracy and computational efficiency.

### 4.1. Benchmark Datasets

#### 4.1.1. Abdominal and Cardiac Segmentation (CT/MRI)

This category represents computationally demanding tasks involving dense volumetric data, serving as the primary testbed for validating Mamba [76,83] and Transformer-based architectures [20,22].

**Synapse Multi-organ Segmentation (Synapse) [218]:** Derived from the MICCAI 2015 challenge, this dataset is the gold standard for evaluating 3D context modeling. It includes 30 CT scans (18 for training, 12 for testing) with annotations for 8 abdominal organs: aorta, gallbladder, spleen, left/right kidneys, liver, pancreas, and stomach.

**Automated Cardiac Diagnosis Challenge (ACDC) [219]:** A cardiac MRI dataset containing 100 exams from patients with various pathologies. It requires segmenting the right ventricle, myocardium, and left ventricle, testing the model’s ability to handle inter-slice consistency in cine-MRI sequences.

**Medical Segmentation Decathlon (MSD) [220]:** A comprehensive benchmark consisting of 10 heterogeneous tasks (Liver, Brain, Hippocampus, Lung, Prostate, Cardiac, Pancreas, Colon, Hepatic Vessel, and Spleen). It is widely used to test the generalization capability of foundation models like MedSAM [9] and nnU-Net [6] baselines.

**AMOS [221]:** A large-scale dataset with 500 CT and 100 MRI scans, annotating 15 abdominal organs. It is increasingly used to validate the robustness of modern large-scale models.

**TotalSegmentator [222]:** A massive CT dataset with 1204 images and 104 anatomical structures, serving as a key pre-training source for foundation models.

#### 4.1.2. Brain Tumor Segmentation (MRI)

The **BraTS (Multimodal Brain Tumor Segmentation)** benchmark series [223] constitutes the *de facto* standard for evaluating 3D volumetric segmentation performance. These datasets provide multi-modal MRI scans (T1, T1ce, T2, FLAIR) and require the segmentation of three nested sub-regions: Whole Tumor (WT), Tumor Core (TC), and Enhancing Tumor (ET).

**BraTS 2019:** Contains 335 training volumes of High-Grade (HGG) and Low-Grade Gliomas (LGG). It serves as a classic baseline for comparing early efficiency-oriented models.

**BraTS 2021:** Represents a significant scale-up, expanding to 1251 training volumes with a focus on glioblastoma. It is currently the most widely used benchmark for training data-hungry architectures like Transformers (e.g., SwinUNETR [57]) and Mamba (e.g., SegMamba [89]).

**BraTS 2023:** Introduces new challenges including Meningioma, Pediatrics, and Missing Modality tasks. This version is particularly relevant for evaluating the generalization capability of Foundation Models and the robustness of Data-Efficient strategies under domain shifts.

#### 4.1.3. Endoscopy, Skin Lesion, and Retinal Segmentation (2D RGB)

These datasets serve as the main arena for *lightweight architectures* (e.g., UNeXt [139], Mobile U-ViT [140]) due to the requirement for real-time inference on edge devices.

**ISIC 2017/2018 [224]:** Large-scale dermoscopy datasets for skin lesion segmentation. The variable lesion shapes and artifacts make them ideal for testing boundary-aware networks like EGE-UNet [148].

**Polyp Segmentation Suite:** Includes *Kvasir-SEG* [225], *CVC-ClinicDB* [226], *CVC-ColonDB*, and *ETIS-Larib*. These contain endoscopic frames of polyps and are used to benchmark real-time performance (FPS) of models like LcmUNet.

**Retinal Vessel Segmentation:** Datasets such as **DRIVE** [227] and **CHASE_DB1** utilize fundus photography for vessel extraction. They evaluate the model’s ability to capture fine-grained, thin tubular structures.

**Cell/Gland Segmentation:** Datasets like **GlaS** [228] and **MoNuSeg** [229] provide histopathology images for gland and nuclei segmentation, testing dense instance prediction capabilities.

**Table 2:** Comprehensive summary of public datasets used in the reviewed papers. (Modality: CT = Computed Tomography, MRI = Magnetic Resonance Imaging, RGB = Optical Image). (Link in table accessed on 13 April 2026).

Dataset	Modality	Dim.	Sample Size	Target Structures	Access Link/Source
*Abdominal & Cardiac (Volumetric)*					
Synapse [218]	CT	3D	30 Vols	8 Organs (Liver, Kidney, Spleen...)	https://www.synapse.org/
ACDC [219]	MRI	3D/4D	100 Patient Exams	RV, Myocardium, LV	https://www.creatis.insa-lyon.fr/Challenge/acdc/
MSD [220]	CT/MRI	3D	2633 Vols (Total)	10 Tasks (Liver, Brain, Lung...)	http://medicaldecathlon.com/
AMOS [221]	CT/MRI	3D	600 Vols	15 Abdominal Organs	https://amos22.grand-challenge.org/
TotalSegmentator [222]	CT	3D	1204 Vols	104 Anatomical Structures	https://github.com/wasserth/TotalSegmentator
LiTS [230]	CT	3D	131 Vols	Liver & Liver Tumor	https://competitions.codalab.org/competitions/17094
KiTS19 [231]	CT	3D	210 Vols	Kidney & Kidney Tumor	https://kits19.grand-challenge.org/
*Brain & Neurological (BraTS Series)*					
BraTS 2019 [223]	MRI (4 modes)	3D	335 Vols	Gliomas (HGG/LGG)	http://braintumorsegmentation.org/
BraTS 2021 [223]	MRI (4 modes)	3D	1251 Vols	Glioblastoma Sub-regions	http://braintumorsegmentation.org/
BraTS 2023 [223]	MRI (4 modes)	3D	2000+ Vols	Glioma, Meningioma, Pediatrics	http://braintumorsegmentation.org/
*Skin, Endoscopy & Retina (2D RGB)*					
ISIC 2018 [224]	Dermoscopy	2D	2594 Images	Skin Lesion	https://challenge.isic-archive.com/data/
Kvasir-SEG [225]	Endoscopy	2D	1000 Images	Polyps	https://datasets.simula.no/kvasir-seg/
CVC-ClinicDB [226]	Endoscopy	2D	612 Images	Polyps	https://polyp.grand-challenge.org/CVCClinicDB/
DRIVE [227]	Fundus	2D	40 Images	Retinal Vessels	https://drive.grand-challenge.org/
GlaS [228]	Histology	2D	165 Images	Glands (Benign/Malignant)	https://warwick.ac.uk/fac/sci/dcs/research/tia/glascontest/
MoNuSeg [229]	Histology	2D	30 Images	Nuclei	https://monuseg.grand-challenge.org/

### 4.2. Evaluation Metrics

Comprehensive evaluation in efficient medical image segmentation necessitates a multi-dimensional analysis, balancing segmentation quality with computational resource consumption. Drawing upon standardized metric protocols [232,233], we categorize evaluation indicators into Segmentation Accuracy Metrics and Computational Efficiency Metrics.

#### 4.2.1. Segmentation Accuracy Metrics

Accuracy metrics quantify the agreement between the predicted segmentation mask *P* and the ground truth annotation *G*.

**Overlap-based Metrics:** These metrics measure the region-wise overlap and are robust to the class imbalance inherent in medical images. **Dice Similarity Coefficient (DSC):** Also known as the F1-score, DSC is the most widely used metric in medical image analysis [232]. It measures the harmonic mean of Precision and Recall:

(17) $$\mathrm{DSC}\left(P,G\right)=\frac{2|P\cap G|}{\left|P|+|G\right|}=\frac{2\mathrm{TP}}{2\mathrm{TP}+\mathrm{FP}+\mathrm{FN}}$$

**Intersection over Union (IoU):** IoU is strictly correlated with DSC but penalizes errors more heavily:

(18) $$\mathrm{IoU}\left(P,G\right)=\frac{\left|P\cap G\right|}{\left|P\cup G\right|}=\frac{\mathrm{DSC}}{2-\mathrm{DSC}}$$

**Pixel-level Classification Metrics:** For binary segmentation tasks like skin lesion segmentation (ISIC dataset), distinguishing between foreground (lesion) and background tissues is critical. These metrics evaluate the model’s pixel-wise classification capability: **Sensitivity (Sen):** Also known as Recall, it measures the proportion of actual positive pixels (lesion) that are correctly identified. High sensitivity is crucial for avoiding missed diagnoses.

(19) $$\mathrm{Sen}=\frac{\mathrm{TP}}{\mathrm{TP}+\mathrm{FN}}$$

**Specificity (Spe):** It measures the proportion of actual negative pixels (background) that are correctly identified. High specificity indicates the model’s ability to reduce false alarms.

(20) $$\mathrm{Spe}=\frac{\mathrm{TN}}{\mathrm{TN}+\mathrm{FP}}$$

**Accuracy (Acc):** It calculates the ratio of correctly classified pixels (both foreground and background) to the total number of pixels.

(21) $$\mathrm{Acc}=\frac{\mathrm{TP}+\mathrm{TN}}{\mathrm{TP}+\mathrm{TN}+\mathrm{FP}+\mathrm{FN}}$$

**Boundary-based Metrics:** In clinical applications such as radiation therapy planning, boundary precision is often more critical than volumetric overlap. **95% Hausdorff Distance (HD95):** The standard Hausdorff Distance (HD) measures the maximum distance between a point in one set and the nearest point in the other. To mitigate the impact of outliers, the 95th percentile (HD95) is widely adopted in benchmarks like MSD and Synapse [220]:

(22) $$\mathrm{HD}95\left(P,G\right)=\max(h_{95}\left(P,G\right),h_{95}\left(G,P\right)),$$

where $h_{95}\left(A,B\right)$ represents the 95th percentile of the distances from points in *A* to their nearest neighbors in *B*. Lower HD95 values indicate better boundary adherence.

#### 4.2.2. Computational Efficiency Metrics

For the *efficient* architectures (e.g., Mamba, Mobile-CNNs), quantifying resource consumption is as vital as accuracy.

**Parameters (Params):** Measured in Millions (M), this metric indicates the model’s spatial complexity and storage requirement. Low-parameter models (e.g., <5 M) are preferred for mobile health applications [139].

**Floating Point Operations (FLOPs):** Measured in Giga-FLOPs (G), FLOPs represent the theoretical computational cost. A key distinction highlighted in this survey is the scaling law: Mamba and CNNs scale linearly (O(N)) with input size *N*, whereas Transformers scale quadratically ($O(N^{2})$) [26].

**Inference Speed (FPS):** Frames Per Second (FPS) measures the practical throughput. Note that low FLOPs do not always guarantee high FPS due to memory access costs (MAC) and hardware optimization levels; thus, reporting FPS on standardized hardware (e.g., NVIDIA A100/RTX 3090) is crucial [119].

**GPU Memory Usage:** Measured in Gigabytes (GB). This metric determines the feasibility of training or deploying models on edge devices. Mamba-based models significantly reduce activation memory compared to Transformers for high-resolution 3D volumes [76].

## 5. Quantitative Analysis and Cross-Category Benchmarking

In this section, we perform a rigorous comparative analysis of the methodologies reviewed in Section 3. To provide a holistic view of the efficiency-accuracy trade-offs, we benchmark representative algorithms from all four defined categories (**State Space Models (Mamba)**, **Efficient Foundation Models (SAM)**, **Lightweight Architectures (CNN/KAN)**, and **Data-Efficient Strategies**) across three mainstream datasets.

It is important to acknowledge that the benchmarking data presented here are synthesized from the results reported in the original literature of each respective methodology. While we aim to provide a comprehensive comparison, this cross-category comparison elucidates the specific strengths and limitations of each paradigm. To ensure academic fairness and reliability, we must discuss potential inconsistencies in experimental settings across different studies—such as variations in GPU architectures, disparate input image resolutions (224×224 vs. 512×512), and different data augmentation protocols. Consequently, these comparisons should be interpreted primarily as performance trend indicators and qualitative trade-offs rather than strict absolute rankings under identical experimental conditions.

### 5.1. Benchmarking on Volumetric Segmentation (Synapse Dataset)

The **Synapse Multi-organ CT dataset** serves as the gold standard for evaluating long-range dependency modeling in dense volumetric data. Table 3 presents a comprehensive comparison of representative methods across different architectural paradigms, including the recently emerged data-efficient strategies.

The experimental results reveal a distinct hierarchy regarding the trade-off between efficiency and accuracy. **Mamba-based architectures** demonstrate superior performance over traditional Transformer baselines. Specifically, **U-Mamba** [76] achieves a high DSC of 85.30% with only 11.85 GFLOPs, significantly outperforming **TransUNet** (77.48% DSC, 88.91 GFLOPs) in both accuracy and computational efficiency. Pure Mamba models like **VM-UNet** [83] further reduce the computational burden to just 4.45 GFLOPs while maintaining a competitive DSC of 81.08%, empirically validating the efficiency of the Selective Scan mechanism for volumetric context modeling. Although **nnFormer** achieves the highest DSC (86.57%), it comes at the cost of a massive parameter count (158.92 M), highlighting Mamba’s advantage in resource-constrained scenarios.

In the realm of **Foundation Model Adaptations**, lightweight adaptations like **SAMed** [234] and **MoE-SAM** [235] show robust performance, with MoE-SAM achieving 84.71% DSC and a remarkably low HD95 of 8.76 mm. However, direct application of heavy backbones, such as in **SAM-Adapter** [236], results in high parameter counts (131.50 M) and suboptimal DSC (72.80%), underscoring the necessity of efficient fine-tuning strategies.

**Lightweight Architectures** continue to evolve, with **CPCANet** [157] achieving 84.48% DSC with moderate parameters (43.43 M), and **SegFormer3D** [165] pushing the limits of efficiency with only 4.50 M parameters while maintaining a DSC of 82.15%. This demonstrates that optimized CNNs and efficient Transformers remain viable for 3D segmentation.

**Table 3:** Cross-category comparison on the **Synapse Multi-organ CT dataset**. Note: For Data-Efficient methods, we report results under limited supervision (10% or 20%) settings.

Category	Method	Backbone	Params (M) ↓	FLOPs (G) ↓	DSC (%) ↑	HD95 (mm) ↓	FPS ↑	GPU
*Baselines*	U-Net [10]	CNN	7.77	13.76	76.85	39.70	39.68	P100
	TransUNet [20]	CNN + ViT	93.23	88.91	77.48	31.69	17.24	A6000
	SwinUNETR [57]	Swin-T	41.25	388.52	79.13	21.55	17.54	A6000
	nnFormer [58]	ViT	158.92	28.62	86.57	10.63	41.67	A6000
**Mamba**	**VM-UNet** [83]	Pure Mamba	27.43	4.45	81.08	19.21	20.61	V100
	**VM-UNet++** [84]	Pure Mamba	52.40	45.21	81.26	20.20	–	L20
	**PHMamba** [237]	Pure Mamba	52.40	45.21	79.86	24.24	–	–
	**U-Mamba** [76]	Hybrid	46.32	11.85	85.30	9.05	–	A100
	**Swin-UMamba** [100]	Hybrid	42.10	10.50	84.70	9.80	–	–
	**MedMamba** [79]	Hybrid	48.50	12.30	80.67	28.15	–	4090
**SAM**	**SAMed** [234]	ViT-B	116.20	44.05	81.88	20.64	–	3090
	**MoE-SAM** [235]	ViT-B	117.81	36.52	84.71	8.76	–	–
	**MedSAM** [9]	ViT-L	73.73	35.63	72.45	20.43	–	–
	**HSAM** [238]	ViT-L	112.30	37.67	86.49	8.18	–	A5000
	**AutoSAM** [129]	ViT-L	93.70	35.02	62.08	27.56	–	A100
	**SAM-Adapter** [236]	ViT-H	131.50	35.32	72.80	33.08	–	A100
**Light**	**ECM-TransUNet** [239]	CNN	66.47	50.68	84.68	15.53	–	4090
	**DLKA-Net** [164]	Hybrid	101.64	19.92	84.27	20.04	–	3090
	**CPCANet** [157]	CNN	43.43	10.62	84.48	15.04	–	3090
	**G-CASCADE** [176]	PVTv2-b2	3.47	1.06	84.54	10.38	–	A6000
	**DAE-Former** [188]	Transformer	48.01	35.00	82.63	16.39	–	3090
	**SegFormer3D** [165]	Efficient ViT	4.50	8.90	82.15	20.50	–	3090
	**KANSeg** [173]	KAN	33.40	9.61	79.95	25.16	–	3090
**Data-Eff**	**RSKD** [240]	ViT	115.35	9.71	82.33	17.21	–	3090
	**MCSCv1** (20%) [194]	CNN	34.80	10.01	68.50	24.80	–	3090
	**MCSCv2Trans** (20%) [194]	Transformer	34.80	10.01	73.10	20.20	–	3090
	**MCSCv1** (10%) [194]	CNN	34.80	10.01	61.10	32.60	–	3090
	**MCSCv2Trans** (10%) [194]	Transformer	34.80	10.01	65.85	23.25	–	3090

Finally, **Data-Efficient Strategies** show promising results under limited supervision. For instance, **RSKD** [240] utilizes knowledge distillation to achieve 82.33% DSC, which is comparable to many fully supervised methods, despite using a Vision Transformer backbone. Semi-supervised approaches like **MCSCv2Trans** [194] demonstrate that with only 20% labeled data, it is possible to achieve a respectable DSC of 73.10%, proving the efficacy of consistency regularization in data-scarce environments.

### 5.2. Benchmarking on Complex Tumor Segmentation (BraTS Dataset)

The **BraTS 2023 MRI dataset** represents the latest and most challenging benchmark for segmenting complex, multi-modal brain tumor sub-regions (Whole Tumor: WT, Tumor Core: TC, Enhancing Tumor: ET). This task is instrumental in evaluating 3D spatial modeling capabilities and generalization across heterogeneous modalities. Table 4 presents comprehensive results covering the four methodological categories: Baseline, Mamba, SAM, Lightweight, and Data-Efficient.

The results on BraTS 2023 highlight the supremacy of 3D-native modeling, particularly Mamba-based architectures. **SegMamba-V2** [85] achieves state-of-the-art performance with an average DSC of 91.60% and the lowest HD95 (3.23 mm). Its improved tri-oriented scanning mechanism captures anisotropic volumetric context effectively, outperforming the original **SegMamba** [89] (91.32% Avg DSC) and **HybridMamba** [102] (91.92% Avg DSC). Notably, all Mamba-based methods significantly surpass traditional Transformer baselines like **SwinUNETR** [57] (88.23% Avg DSC) and **nnFormer** [58] (85.27% Avg DSC), demonstrating the efficiency of linear complexity in modeling long-range dependencies in 3D volumes.

In the realm of **Foundation Models**, adaptations like **MSCG** [242] demonstrate robust performance with an average DSC of 87.19%, surpassing the standard **MedSAM** [9] (85.98%) and the original **SAM** [27] (83.01%). This improvement suggests that specialized adaptation layers are crucial for transferring SAM’s capabilities to the medical domain. However, these models generally trail behind Mamba-based architectures in terms of boundary precision (HD95), likely due to the inherent domain shift and the computational constraints of adapting heavy 2D backbones to 3D tasks.

For **Lightweight Architectures**, **ECM-TransUNet** [239] achieves a remarkable HD95 of 2.27 mm and a high DSC of 90.75%, proving that optimized lightweight designs can rival heavy baselines in boundary delineation. In contrast, **3D ST-Net** [244] shows lower performance (81.80% Avg DSC) with significantly higher HD95, indicating the difficulty of balancing lightweight design with the complex spatial modeling required for brain tumors.

Regarding **Data-Efficient Strategies**, methods trained with limited supervision, such as **WeaklySeg** [245] and **SelfBLLP** [246], understandably lag behind fully supervised methods. **TransferSeg** [247] achieves an average DSC of 76.31%, highlighting the challenge of learning robust features from scarce data in complex 3D environments compared to the rich supervision used in Mamba and SAM approaches.

### 5.3. Benchmarking on 2D Skin Lesion Segmentation (ISIC Dataset)

The **ISIC 2018 Skin Lesion dataset** serves as a representative benchmark for 2D medical image segmentation. Unlike volumetric data, dermoscopy images require models to handle high-resolution texture details and variable lesion shapes, often in the presence of artifacts. In this track, we benchmark representative models from **Mamba Architectures**, **Efficient Foundation Models**, **Lightweight Architectures**, and **Data-Efficient Strategies**. Table 5 details the comparison across clinical metrics including Dice Similarity Coefficient (DSC), Sensitivity (Sen), Specificity (Spe), and Accuracy (Acc).

Mamba-based architectures demonstrate superior capability in modeling global context compared to traditional CNN and Transformer baselines in 2D scenarios. Notably, **CCViM** [248], which utilizes context clustering within the SSM framework, achieves a DSC of 90.06%, outperforming both **U-Net** (86.51%) and the hybrid **TransUNet** (88.12%). Similarly, **VM-UNet++** [84] achieves consistent improvements across all metrics (89.86% DSC) by leveraging nested skip connections with visual state space blocks, proving that SSMs effectively capture the long-range dependencies required to delineate irregular lesion boundaries.

Among the evaluated categories, Foundation Model adaptations exhibit the highest performance ceiling on this dataset. **Med-SA** [250] and **LoRA-MedSAM** [251] achieve remarkable DSC scores of 97.06% and 97.02%, respectively. This indicates that pre-trained knowledge, when efficiently adapted via shuffle attention or low-rank adaptation, significantly enhances feature discrimination for skin lesion segmentation, far surpassing models trained from scratch. However, these models typically require higher computational resources compared to purpose-built lightweight networks.

In the lightweight category, **MAUNext** [153] stands out with a DSC of 90.52%, which is competitive with, or even superior to, larger Mamba-based models. **LeaNet** [155] also maintains a high specificity of 97.72%, proving that streamlined channel attention mechanisms can effectively balance segmentation accuracy and computational efficiency for edge deployment.

Under limited supervision settings, Mamba-based semi-supervised frameworks show promise. **DCMamba** [111] consistently outperforms **Semi-Mamba-UNet** [29] at both 10% and 20% labeling ratios. For instance, with only 20% labeled data, DCMamba achieves 86.10% DSC, approaching the performance of fully supervised CNN baselines (U-Net), thereby validating the effectiveness of diversity-enhanced consistency learning in data-scarce scenarios.

### 5.4. Summary and Guidelines for Model Selection

Synthesizing the extensive cross-category benchmarking results presented in Table 3, Table 4 and Table 5, we derive evidence-based guidelines for model selection tailored to specific clinical scenarios. The trade-offs between segmentation accuracy (DSC), computational complexity (FLOPs), and resource constraints (Memory/Params) indicate that no single architecture dominates all domains. Instead, the optimal choice depends heavily on the specific application constraints:

**For High-Precision Volumetric Analysis (CT/MRI): Hybrid Mamba Architectures** (e.g., U-Mamba [76], SegMamba-V2 [85]) emerge as the optimal choice. As evidenced in Section 5.1 (Synapse) and Section 5.2 (BraTS), these models achieve state-of-the-art accuracy (DSC >86% on Synapse, >91% on BraTS) while maintaining linear computational complexity. By successfully circumventing the quadratic memory bottlenecks inherent in Transformers, they enable the efficient processing of high-resolution 3D volumes on standard GPUs without introducing patch-wise artifacts, effectively solving the long-range dependency problem that limits traditional CNNs.

**For Resource-Constrained Edge Deployment:** For 2D applications on mobile or point-of-care devices, **Lightweight Architectures** demonstrate superior efficiency. As shown in Section 5.3 (ISIC), models like **MAUNext** [153] and **LeaNet** [155] offer the best balance between high inference throughput and segmentation precision. Additionally, **Mamba-based lightweight variants** (e.g., LightM-UNet [105]) are preferred when capturing global context is critical even on edge devices, outperforming traditional CNNs in complex boundary delineation.

**For Generalization and Data-Scarce Scenarios:** When addressing unseen tasks or operating under limited supervision, **Adapted Foundation Models** and **Data-Efficient Strategies** prove superior. In the BraTS benchmark (Section 5.2), adapters like MSCG [242] leverage pre-trained knowledge to ensure robust generalization across complex modalities, making them indispensable for tasks with limited annotations. Furthermore, in scenarios requiring privacy preservation, federated learning frameworks such as FedDP [30] demonstrate that decentralized training can achieve performance comparable to centralized baselines (88.45% DSC), offering a viable path for multi-center collaboration without compromising patient data privacy.

In summary, the field is transitioning from a “one-size-fits-all” approach dominated by U-Net to a diverse ecosystem where **Mamba** addresses the computational limits of 3D modeling, **Foundation Models** solve the generalization puzzle, and **Lightweight Architectures** redefine efficiency for edge deployment.

## 6. Challenges and Future Directions

While the transition from CNNs and Transformers to Mamba, Foundation Models, and KANs has significantly advanced the efficiency of medical image segmentation, the pursuit of low computational cost must not compromise clinical reliability. Building upon the taxonomy established in Section 3 and the benchmarking results in Section 5, we identify four critical frontiers where efficiency intersects with theoretical robustness, generative capability, and system-level collaboration.

**Theoretical Robustness of Linear Sequence Modeling:** The primary motivation for adopting State Space Models (Section 3.1) is their linear computational complexity. However, the theoretical stability of these models when processing extremely long biomedical sequences, such as whole-body CT scans or high-resolution histopathology slides, remains an open challenge. Although Mamba’s selective scan mechanism effectively filters noise, it may inadvertently suppress spatially distant but semantically critical pathological details, a phenomenon known as “long-term forgetting”. To address this, researchers are exploring concrete architectural remedies, such as MemMamba [252], which incorporates an explicit memory storage to decouple sequence length from hidden state retention, and Block-Biased Mamba [253], which enhances local dependency preservation through localized scan biases. Furthermore, hybrid architectures that interleave selective SSMs with periodic self-attention layers are gaining traction as they leverage attention-based retrieval to “refresh” the hidden state, ensuring that distal anatomical context is not decayed. Furthermore, rigorous analysis of Mamba-based architectures under adversarial attacks or domain shifts is essential, as current works like U-Mamba [76] primarily optimize for standard segmentation metrics rather than safety-critical robustness.

**Generative and Interactive Segmentation Paradigms:** Current efficient adaptations of Foundation Models (Section 3.2) predominantly operate in a discriminative manner, outputting deterministic masks. However, medical imaging is inherently ambiguous, necessitating the quantification of uncertainty. A promising frontier is the integration of efficient backbones into Generative Segmentation, particularly Diffusion Models. Recent innovations like MambaDiff [254] and Prior-Guided Residual Diffusion [255] replace heavy U-Net denoisers with linear-complexity SSMs, significantly accelerating the sampling process. Simultaneously, enhancing interactivity is crucial. Future systems should evolve beyond geometric prompts to leverage Language-Vision Models (LVMs) for semantic interactivity, as pioneered by MedCLIP-SAM [133]. The ultimate goal is to construct efficient “Context-Aware Foundation Models” that reason about patient history and textual reports, shifting from simple pixel-level segmentation to holistic clinical understanding.

**Explainability in Lightweight Architectures:** The emergence of KANs and tokenized MLPs (Section 3.3) has pushed parameter efficiency to new limits. However, as models become more compact and abstract, their decision-making processes often become opaque “black boxes”, hindering clinical trust. KANs offer a unique opportunity to bridge this gap by learning symbolic functions (splines) on edges rather than fixed weights. Future research should exploit this property to develop Explainable Efficient AI, where models like KM-UNet [171] not only output segmentation masks but also visualize the mathematical basis of their decisions. Aligning architectural efficiency with the interpretability standards discussed in trustworthy AI reviews [256] is essential for regulatory approval.

**Edge-Cloud Collaboration and Continuous Learning:** Finally, the data-efficient strategies reviewed in Section 3.4 point towards a systemic evolution: Edge-Cloud Collaboration. While lightweight models like Mobile U-ViT [140] are optimized for inference on edge devices (e.g., portable ultrasound probes), they lack the capacity to learn from vast, diverse datasets. A future trend will likely involve a decoupled learning paradigm: heavy foundation models reside on the cloud to aggregate global knowledge via Federated Learning, while lightweight student models are continuously updated and deployed to the edge via Knowledge Distillation. This framework requires novel algorithms for bandwidth-efficient model synchronization, such as FKD-Med [207], and continuous learning mechanisms that adapt to new imaging modalities without catastrophic forgetting, a capability where the stateful nature of Mamba may offer distinct advantages over stateless architectures.

**Translational and Regulatory Barriers for Efficient AI:** Despite the algorithmic advancements, the clinical transition of these efficient models faces significant translational and regulatory hurdles. Current regulatory frameworks, such as those established by the FDA and CE, typically require “locked” model versions to ensure safety and reproducibility. However, the dynamic nature of the paradigms discussed—specifically Parameter-Efficient Fine-Tuning (PEFT) and Federated Learning—creates a “moving target” for certification, as models are designed to evolve with new local data. Furthermore, practical deployment requires high robustness against clinical artifacts (e.g., motion blur or sensor drift) that are not always present in curated benchmarks. To provide a complete roadmap for clinical adoption, future research must prioritize the development of standardized validation protocols for continuously learning systems and ensure that efficiency-driven architectures maintain high stability under diverse, real-world deployment scenarios.

## 7. Conclusions

This survey elucidates the critical paradigm shift in medical image segmentation, transitioning from an era dominated by accuracy-centric architectures to one prioritizing computational and data efficiency. Through a systematic review of the latest literature, we established a comprehensive taxonomy encompassing four pivotal streams: State Space Models (Mamba), Efficient Foundation Model Adaptation, Advanced Lightweight Architectures, and Data-Efficient Strategies. Our analysis highlights that Mamba-based frameworks successfully circumvent the quadratic complexity limitations of Vision Transformers, establishing a new standard for high-resolution volumetric analysis. Meanwhile, efficient adaptations of Foundation Models bridge the divide between large-scale pre-training and clinical applicability, enabling robust zero-shot generalization with reduced resource demands. Furthermore, the renaissance of large-kernel CNNs and the advent of KANs have redefined parameter efficiency for edge computing, complemented by data-efficient strategies that effectively alleviate annotation scarcity and address privacy concerns. Collectively, these technological advancements propel the democratization of medical AI. By dismantling computational barriers and reducing dependence on extensive annotations, the field is poised to deliver next-generation segmentation systems that achieve an optimal balance of accuracy, efficiency, and robustness, ensuring their broad deployability across diverse clinical environments.

## Figures and Tables

**Table 1:** Summary of representative efficient medical image segmentation methods (2022–2025). Note: SSM = State Space Model, KD = Knowledge Distillation, PEFT = Parameter-Efficient Fine-Tuning, FL = Federated Learning, UDA = Unsupervised Domain Adaptation.

Category	Sub-Category	Representative Methods	Core Mechanism/Innovation	Year
**Mamba & SSM**	Pure Mamba	U-Mamba [76], U-RWKV [82]	SS2D Scanning, RWKV Architecture	2024–2025
	Hybrid CNN-Mamba	SegMamba [89], MambaVesselNet++ [90]	Gated Spatial Conv, Mamba-Decoder	2024–2025
	Hybrid ViT-Mamba	Swin-UMamba [100], HMT-UNet [101]	Linear Complexity Self-Attention Replacement	2024–2025
	Advanced Scanning	Switch-UMamba [86], LoG-VMamba [87]	Dynamic Routing, Local-Global Scan	2024–2025
**Efficient SAM**	Adapters (PEFT)	Mamba-SAM [117], MA-SAM [113]	Mamba/Low-Rank Adapters	2024
	Distillation	EfficientMedSAM [119], KD-MedSAM [120]	NAS + Distillation, Lightweight Student	2024–2025
	Auto-Prompting	AutoSAM [129], MedCLIP-SAM [133]	Prompt Generator, Text-Visual Alignment	2023–2024
**Lightweight**	Mobile/MLP	UNeXt [139], Mobile U-ViT [140]	Tokenized MLP, Inverted Bottleneck	2022–2025
	Large Kernel	3D UX-Net [161], CMUNeXt [163]	Large Kernel Depthwise Conv (7×7+)	2023–2024
	KAN	U-KAN [170], KM-UNet [171]	Kolmogorov-Arnold Networks (Splines)	2025
**Data-Efficient**	Semi-Supervised	SSL4MIS [190], Semi-Mamba [29]	Cross-Teaching, Consistency Reg.	2022–2024
	Federated Learning	FedDP [30], FKD-Med [207]	Personalized FL, Distillation-based Comm.	2024
	Domain Adapt.	LE-UDA [212], Mamba-UDA [211]	Adversarial Alignment, SSM for Domain Inv.	2023–2025

**Table 4:** Comprehensive cross-category comparison on the **BraTS 2023 MRI dataset**.

Category	Method	WT		TC		ET		Avg	
		DSC (%)↑	HD95 (mm)↓	DSC (%)↑	HD95 (mm)↓	DSC (%)↑	HD95 (mm)↓	DSC (%)↑	HD95 (mm)↓
**Baseline**	UNetr [241]	92.19	6.17	86.39	5.29	84.48	5.03	87.68	5.49
	SwinUNETR [57]	92.71	5.22	87.79	4.42	84.21	4.48	88.23	4.70
	nnFormer [58]	91.15	5.65	85.94	5.31	78.73	5.09	85.27	5.35
**Mamba**	**SegMamba** [89]	93.61	3.37	92.65	3.85	87.71	3.48	91.32	3.56
	**SegMamba-V2** [85]	94.02	3.41	92.83	2.92	87.93	3.36	**91.60**	3.23
	**HybridMamba** [102]	94.10	3.78	92.84	3.30	88.83	3.35	91.92	3.48
	**U-Mamba** [76]	91.62	4.31	90.17	5.23	86.03	4.86	89.27	4.80
**SAM**	**SAM** [27]	87.66	13.98	84.88	9.67	76.47	8.40	83.01	10.68
	**MedSAM** [9]	89.46	4.85	86.75	6.07	81.22	3.90	85.98	4.94
	**MSCG** [242]	91.53	4.68	89.12	5.87	80.94	3.97	87.19	4.84
	**SAM2** [243]	88.95	7.45	86.13	6.30	80.31	5.72	85.13	6.49
**Light**	**ECM-TransUNet** [239]	–	–	–	–	–	–	90.75	**2.27**
	**3D ST-Net** [244]	85.99	80.50	78.90	25.71	39.36	40.44	81.80	35.17
**Data-Eff**	**WeaklySeg** [245]	–	–	–	–	–	–	74.50	20.80
	**SelfBLLP** [246]	82.68	22.85	74.06	29.22	59.63	121.02	72.12	57.70
	**TransferSeg** [247]	80.90	40.40	76.72	26.76	71.30	43.91	76.31	37.02

**Table 5:** Cross-category comparison on the **ISIC 2018 (2D Skin Lesion)** dataset. Note that Data-Efficient methods are trained with limited supervision (10% or 20%).

Category	Method	Core Mechanism	DSC (%) ↑	Sen (%) ↑	Spe (%) ↑	Acc (%) ↑
**Baseline**	U-Net [10]	Standard CNN	86.51	88.69	94.90	92.91
	TransUNet [20]	CNN + ViT	88.12	89.40	94.04	93.91
**Mamba**	**UD-Mamba** [109]	Uncertainty-Driven	89.15	89.55	96.26	94.60
	**Swin-UMamba** [100]	Hybrid Swin-Mamba	88.60	89.21	95.87	94.53
	**VM-UNet++** [84]	Nested Skip	89.86	90.05	96.66	95.05
	**CCViM** [248]	Context Clustering	90.06	88.74	97.32	95.23
**SAM**	**MedSAM** [9]	Full Fine-tuning	94.86	–	–	98.48
	**I-MedSAM** [249]	Adapter Tuning	95.39	–	–	98.53
	**Med-SA** [250]	Shuffle Attention	97.06	–	–	98.95
	**LoRA-MedSAM** [251]	Low-Rank Adapt	97.02	–	–	98.74
**Light**	**MAUNext** [153]	Multi-scale Attn	90.52	88.80	–	93.83
	**LeaNet** [155]	Efficient Channel Attn	88.89	90.63	97.72	95.72
**Data-Eff**	**DCMamba** (10%) [111]	Diversity SSL	85.45	87.59	96.27	93.73
	**DCMamba** (20%) [111]	Diversity SSL	86.10	87.99	95.85	93.98
	**Semi-Mamba** (10%) [29]	Cross-Supervised	81.74	85.11	94.86	92.32
	**Semi-Mamba** (20%) [29]	Cross-Supervised	84.09	86.11	95.41	93.11

## Data Availability

The datasets analyzed in this study are publicly available, and their sources are provided in the manuscript. All relevant links to the datasets are included in the corresponding sections of the paper.
